# Nontuberculous mycobacteria testing and culture positivity in the United States

**DOI:** 10.1186/s12879-024-09059-9

**Published:** 2024-03-06

**Authors:** Julia E. Marshall, Rachel A. Mercaldo, Ettie M. Lipner, D. Rebecca Prevots

**Affiliations:** Epidemiology and Population Studies Unit, Division of Intramural Research, National Institute of Allergy and Infectious Diseases, National Institutes of Health, 5601 Fishers Ln, Bethesda, MD 20852 USA

**Keywords:** Nontuberculous mycobacteria, Prevalence, Acid-fast bacilli testing, Mycobacterium avium complex, Mycobacterium abscessus

## Abstract

**Background:**

Nontuberculous mycobacteria (NTM) are environmental bacteria which may cause chronic lung disease. The prevalence of NTM pulmonary infection and disease has been increasing in the United States and globally. The predominant clinically relevant species of NTM in the United States are *Mycobacterium avium* complex (MAC) species and *Mycobacterium abscessus*. With the development of rapid species identification methods for NTM (e.g. PCR probes), more testing for NTM is being conducted through commercial labs, such as Laboratory Corporation of America (Labcorp), which provides deidentified real-time testing data to the Centers for Disease Control (CDC) pursuant to a data sharing agreement. Because NTM lung infections are not reportable in most states, other data sources are key to understanding NTM testing patterns, positivity rates, and species distributions to track infection trends and identify clinical care needs.

**Methods:**

We obtained national Labcorp data for the period January 2019 through mid-April 2022. We subset the data to only respiratory samples sent for Acid Fast Bacilli (AFB) cultures. NTM positive results were defined as those which identified an NTM species and are not *Mycobacterium tuberculosis*, *Mycobacterium bovis*, or *Mycobacterium gordonae*.

**Results:**

Overall, 112,528 respiratory samples were sent for AFB testing during the study period; 26.3% were from the Southeast U.S., identified as HSS Region IV in the Labcorp dataset, and 23.0% were from the Pacific and South Pacific region (Region IX). The culture positive prevalence ranged from 20.2% in the Southeast to 9.2% in the East North Central region (Region V). In the Southeast US, *M. abscessus* prevalence was 4.0%. For MAC, the highest prevalence was observed in the Mountain region (Region VII) (13.5%) and the lowest proportion was in the East South Central region (7.3%, Region III). Among positive tests, the proportion which was MAC varied from 61.8% to 88.9% and was highest in the Northeast U.S. The proportion of positive samples which were M. *abscessus* ranged from 3.8% to 19.7% and was highest in the Southeast.

**Conclusions:**

The Southeastern region of the U.S. has the highest rate of culture positivity in Labcorp tests for total NTM and, of all positive tests, the highest proportion of *M.* *abscessus*. These estimates may underrepresent the true number of *M.* *abscessus* infections because *M*. *absesscus*-specific probes are not commercially available and not all NTM testing in the United States is done by Labcorp. Analysis of real-time testing data from commercial laboratories may provide insights into risk factors for NTM culture positivity in ‘hotspot’ areas.

**Supplementary Information:**

The online version contains supplementary material available at 10.1186/s12879-024-09059-9.

## Background

Nontuberculous mycobacteria (NTM) are highly prevalent in the environment, and pathogenic species can cause chronic lung disease [1–3]. Two highly prevalent, clinically relevant species in the United States (U.S.) are *Mycobacterium avium* complex (MAC) species and *Mycobacterium abscessus species*. Exposure to pathogenic NTM species occurs primarily through drinking water, soil, and aerosols [1]. NTM risk and species-specific trends vary across the United States (U.S.) [4–7], but overall infection and disease incidence and prevalence are increasing in the United States [3, 8]. This geographic variation is associated with differences in environmental factors [7, 9–14]. Some states, such as Florida and Hawaii, have been identified as NTM ‘hotspots’ [7, 15, 16]. NTM incidence and prevalence are increasing across U.S. regions [2, 3, 8].

Population-based data for pulmonary NTM infection and disease prevalence in the general population are limited because fewer than 20 states include NTM infection as a reportable condition [17]. Of the states that collect data on NTM, few differentiate between extrapulmonary and pulmonary NTM or collect species data [17]. NTM species identification is critical to informing clinical care because different species of NTM require different course of treatment [18]. Moreover, different species have different environmental niches whose understanding can guide mitigation strategies [19].

Due to the increasing accessibility of next-generation sequencing technologies, a number of culture-independent methods of NTM detection have been developed and are increasingly used [20]. Culture-independent methods of NTM species identification can be used for rapid diagnostic testing in resource-limited locations [20]. Traditional culturing methods of NTM identification can take weeks to grow NTM and some species are difficult to detect using these methods [20]. The development of commercially available PCR probes for NTM has facilitated an increase in NTM testing by commercial laboratories, such as Labcorp.

Beginning in 2020, Labcorp, one of the largest clinical laboratory networks in the U.S., and the Center for Disease Control’s (CDC) National Syndromic Surveillance Program (NSSP) initiated a data sharing agreement as part of the response to the COVID-19 pandemic [21]. Through this agreement, Labcorp has shared testing data from its thousands of locations in the U.S. with the NSSP in real-time. This data sharing agreement offers an unprecedented opportunity to evaluate the current role of commercial laboratories in NTM testing in the U.S. The objective of this study was to describe the NTM culture positivity and proportionate-species distributions in U.S. Health and Human Services (HHS) regions using Labcorp data.

## Methods

We obtained data through the CDC’s National Syndromic Surveillance Program for all acid-fast bacilli (AFB) tests administered by Labcorp from January 2019 to April 2022. This study was designated as “Not Research—Public Health Surveillance” by the Office of Management and Budget. All test results were deidentified to maintain subject confidentiality, thus this analysis was conducted at the test level. Geographic location of sample collection was available at the HHS region resolution (See Appendix 1 for more information on HHS regions).

**Fig. 1:**
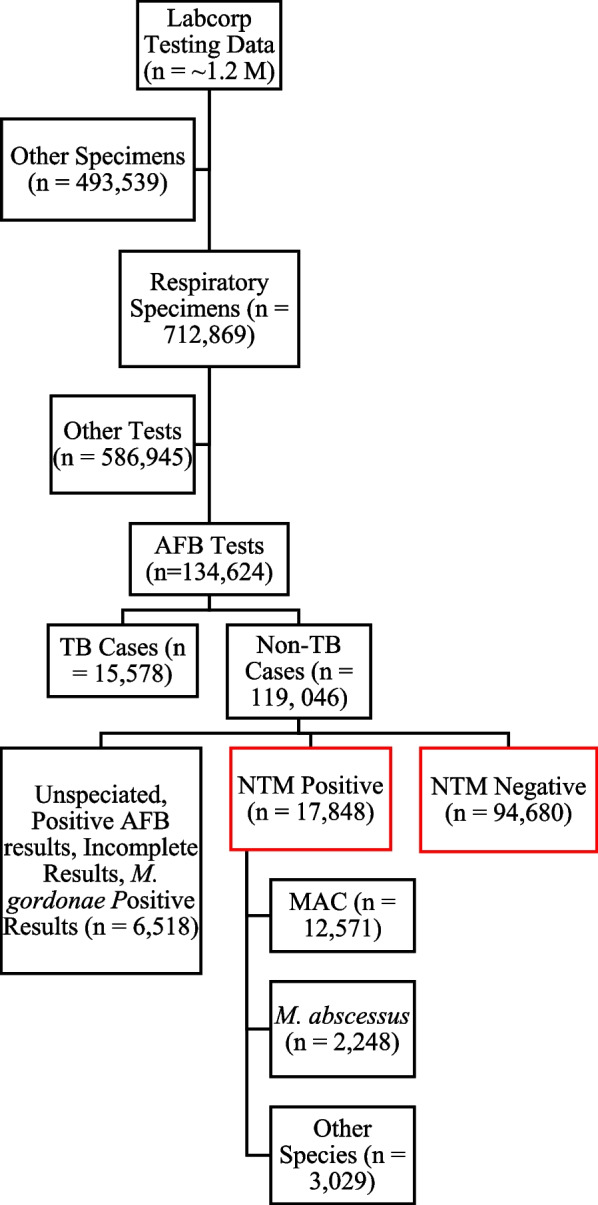
Workflow of study population identification. Red color indicates groups analyzed in this study

We excluded all AFB tests on non-respiratory specimens or with missing specimen information. We identified respiratory specimens using text searches of specimen data. Respiratory specimens that could not be identified using this approach were identified using International Classification of Diseases, Tenth Revision (ICD-10) codes associated with respiratory conditions (Fig. 1). All AFB tests on a sample from a respiratory site served as the denominator. Data on mycobacterial culture speciation was stored in a text field. We analyzed the frequency of all species terms and detected no spelling or other entry errors for the species of interest. We then extracted all instances of MAC and *M. abscessus* by searching the text for “mac”, “avium”,”intracellulare”, and “abscessus” using regular expressions, via the R base package function *regex*. We defined positive NTM tests as tests that identified a NTM species and were not identified as *M. tuberculosis*, *M. tuberculosis complex, M. gordonae,* or *M. bovis*. We defined negative NTM tests as AFB tests that indicated no AFB species were detected. AFB tests that did not identify an NTM species, lacked results, or used *Mycobacteria tuberculosis*-specific tests were excluded. We calculated the AFB testing rate by region, NTM culture positivity prevalence, and the proportionate-species distributions by HHS region.

## Results

Labcorp received 112,528 respiratory samples for AFB testing between January 2019 and April 2022. We identified 17,848 (15.9%) positive NTM results. Of these results, 12,571 (70.4%) were MAC, 2,248 (12.6%) were *M. abscessus*, and 3,029 (17.0%) were other species. Age and sex of persons from whom samples were collected for AFB testing are summarized by NTM species in Table 1.

**Table 1:** Demographic characteristics of persons tested for AFB by Labcorp by test and NTM species (excluding *M. gordonae*)

Variable	Total AFB	Total NTM	MAC	*M. abscessus*	Other Species
Results (N (% tests/% NTM positives)	112,528	17,848 (15.9/100)	12,571 (11.2/70.4)	2248 (2.0/12.6)	3029 (2.7/17.0)
Female (%)	52.8	59.8	61.8	59.5	51.6
Age (Mean)	64.3	68.5	69.1	67.0	67.4

**Fig. 2:**
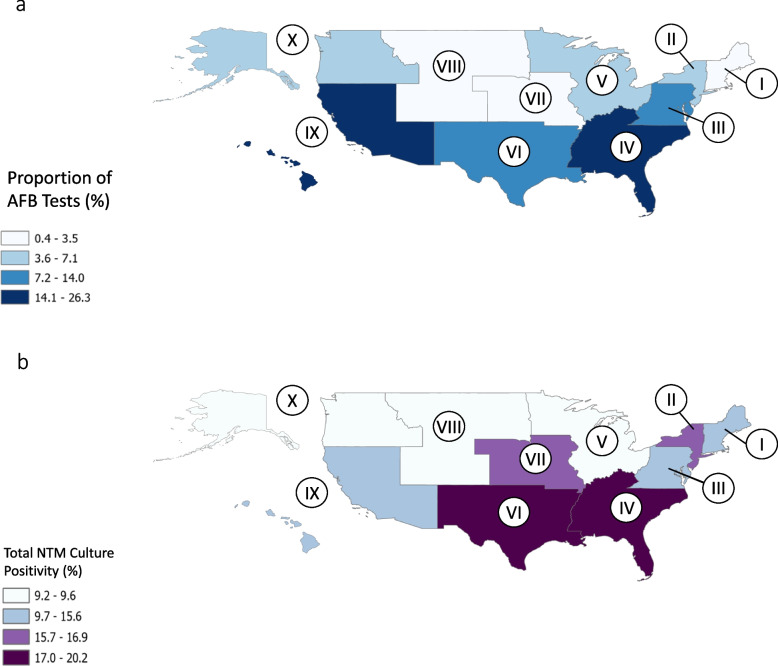
Categories of culture positivity correspond to quantiles of data. The HHS regions represent the states as follows: Region I (CT, ME, MA, NH, RI, VT), Region II (NJ, NY, PR, VI), Region III (DC, DE, MD, VA, WV, PA), Region IV (KY, TN, NC, SC, GA, AL, MS, FL) Region V (MN, WI, MI, IL, IN, OH), Region VI (NM, TX, OK, AR, LA), Region VII (NE, IA, KS, MO), Region VIII (ND, SD, MT, WY, CO, UT), Region IX (CA, NV, AZ, HI, GU, AS, MP, PW, FM, MH), Region X (AK, WA, OR, ID). **a** Proportion of Labcorp Tests Administered by Region. **b** Total NTM Culture Positivity (excluding *M. gordonae*)

AFB testing volume by Labcorp varied by region, with the highest proportion of AFB tests from the Southeast U.S. (KY, TN, NC, SC, GA, AL, MS, FL) (26.3%), represented by Region IV in the Labcorp data set, followed by the Pacific and South Pacific (Region IX, CA, NV, AZ, HI, GU, AS, MP, PW, FM, MH) (23.0%) and the West South Central (Region VI, NM, TX, OK, AR, LA) (14.0%) (Fig. 2a). The lowest proportion of AFB tests were from the Northeast U.S. (Region I, CT, ME, MA, NH, RI, VT) (0.37%), the Mountain region (Region VII, NE, IA, KS, MO) (1.1%), and the West North Central (Region VIII, ND, SD, MT, WY, CO, UT) (3.5%) (Fig. 2a).

NTM isolation prevalence ranged from 9.2% in the East North Central U.S. (Region V, MN, WI, MI, IL, IN, OH) to 20.2% in the Southeastern U.S. (Fig. 2b). For MAC, the region with the highest isolation prevalence was in the Mountain region (13.5%), the Middle Atlantic and Atlantic (Region II, NJ, NY, PR, VI) (12.8%), and the Southeast U.S. (12.5%). MAC isolation prevalence was lowest in the East North Central U.S. (7.3%,). For *M. abscessus,* isolation prevalence was highest in the Southeast U.S. (4.0%) and lowest in the West North Central region (0.36%).

**Fig. 3:**
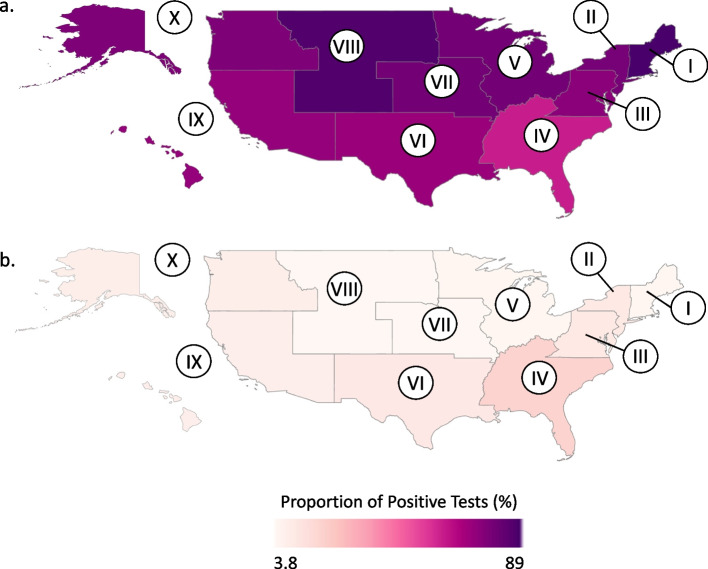
The HHS regions represent the states as follows: Region I (CT, ME, MA, NH, RI, VT), Region II (NJ, NY, PR, VI), Region III (DC, DE, MD, VA, WV, PA), Region IV (KY, TN, NC, SC, GA, AL, MS, FL) Region V (MN, WI, MI, IL, IN, OH), Region VI (NM, TX, OK, AR, LA), Region VII (NE, IA, KS, MO), Region VIII (ND, SD, MT, WY, CO, UT), Region IX (CA, NV, AZ, HI, GU, AS, MP, PW, FM, MH), Region X (AK, WA, OR, ID). **a** Proportion of positive tests (excluding *M. gordonae*) identified as MAC. **b** Proportion of positive tests (excluding *M. gordonae*) identified as *M. abscessus*

We were also interested in the proportion of positive NTM tests that were MAC or *M. abscessus*, within each region. The proportion of positive tests identified as MAC was highest in the following regions: Northeast (88.9%), West North Central (86.8%), and Mountain (79.9%) (Fig. 3a). MAC relative proportions were lower in the Southeast (61.8%), the West South Central (71.8%), and the Pacific and South Pacific regions (72.9%) (Fig. 3a). The relative proportion of *M. abscessus* was highest in the Southeast (19.7%), the West South Central (10.9%), and the Middle Atlantic and Atlantic regions (10.4%) (Fig. 3b). The proportion *M. abscessus* was lowest in the Mountain region (3.8%) (Fig. 3b). Other NTM species represented between 6.7% (Northeast) and 19.2% (Pacific and South Pacific) of positive NTM cultures.

## Discussion

We found that during the study period April 2019 through 2022 the prevalence of all NTM and *M. abscessus* isolates in respiratory cultures was highest in the Southeast U.S. We also found this region had the highest proportion of positive tests identified as *M. abscessus*. These findings are consistent with prior work which identified the southeastern states, namely Florida, as high-risk for NTM and as having a high burden of *M. abscessus* in the general population and in high-risk groups [4, 7–9, 16, 22, 23]. This work highlights the value of real-time data on NTM culture results, which can inform public health action and clinical needs planning.

We found the proportion of positive tests that were MAC ranged from 61.8% to 88.9% and was highest in the Northeast U.S. This finding is consistent with prior studies in which MAC represented 61%-78% of all species identified in U.S. Census regions [24]. This study identified the East South Central Region (included in the Southeast) and the Northeast as having the highest proportion of positive MAC cultures in the U.S. [24]. This is consistent with our prior study, which found the Northeast had the highest burden of MAC in the cystic fibrosis population [8].

The regional, species-specific trends observed in this study support the validity of NTM reporting from respiratory isolates through commercial laboratories. Many patients experience multi-year delays in diagnosis, which is difficult due to the general symptoms associated with disease and the clinical, microbiologic, and radiologic diagnostic criteria [18, 25]. Due to these challenges, NTM microbiological data is frequently used in epidemiologic analyses of NTM in the U.S. [12, 26]. At present, NTM is not a reportable disease in most states. Of the 14 states that report NTM in some form, 4 report only extrapulmonary, 2 report extrapulmonary and pulmonary NTM, and 8 report NTM but do not specify specimen type or NTM species [17]. Commercial laboratory networks, such as Labcorp, offer the possibility of analyzing NTM testing data in real-time. Future research should evaluate the population served by various commercial laboratories across the U.S.

This study has some important limitations. The dataset used in this analysis is not population-based. Labcorp NTM testing volumes vary by region (Fig. 2a). Data on the population served by Labcorp is not available at present, nor is longitudinal NTM testing data. Because of the low prevalence of tuberculosis in the United States, these data were filtered only for *M. tuberculosis, M. bovis*, and *Mycobacterium tuberculosis* complex positive results [27]. Our results likely underestimate the true *Mycobacterium tuberculosis* complex and *M. abscessus* prevalence. PCR probes are not commercially available for *M. abscessus* at present. *M. abscessus* identification requires lengthy culture protocols, while PCR probes are commercially available for MAC species and enable rapid detection. Despite these considerations, the regional patterns of MAC and *M. abscessus* burden align with previously observed patterns.

## Conclusions

The southeastern region of the U.S. states had the highest total NTM and *M. abscessus* respiratory culture positivity prevalence. The largest proportion of Labcorp AFB testing of respiratory specimens occurred in the southeastern region as well. Real-time disease data of nonreportable diseases, such as pulmonary NTM, collected by commercial laboratories may inform identification of case clusters and risk factors.

### Supplementary Information

**Additional file 1:** **Supplemental Figure 1.**

## Data Availability

The data that support the findings of this study are not publicly available because they were obtained from the National Syndromic Surveillance Program of the Centers for Disease Control and Prevention through a Data Use Agreement. All access to NSSP data requires active, documented collaboration with an NSSP–ESSENCE or CDC user. For approval guidelines, please email NSSP@cdc.gov.

## Appendix 1

Language Used to Describe HHS Regions

Northeast: Region I (CT, ME, MA, NH, RI, VT).

Middle Atlantic and Atlantic: Region II (NJ, NY, PR, VI).

East South Central: Region III (DC, DE, MD, VA, WV, PA).

Southeast: Region IV (KY, TN, NC, SC, GA, AL, MS, FL).

East North Central: Region V (MN, WI, MI, IL, IN, OH).

West South Central: Region VI (NM, TX, OK, AR, LA).

Mountain: Region VII (NE, IA, KS, MO).

West North Central: Region VIII (ND, SD, MT, WY, CO, UT).

Pacific and South Pacific: Region IX (CA, NV, AZ, HI, GU, AS, MP, PW, FM, MH).

North Pacific: Region X (AK, WA, OR, ID).
