# Correction: Nontuberculous mycobacteria testing and culture positivity in the United States

**DOI:** 10.1186/s12879-024-09216-0

**Published:** 2024-04-10

**Authors:** Julia E. Marshall, Rachel A. Mercaldo, Ettie M. Lipner, D. Rebecca Prevots

**Affiliations:** Epidemiology and Population Studies Unit, Division of Intramural Research, National Institute of Allergy and Infectious Diseases, National Institutes of Health, 5601 Fishers Ln, Bethesda, MD 20852 USA

**Correction** **:** **BMC Infect Dis 24, 288 (2024)**

**https://doi.org/10.1186/s12879-024-09059-9**

Following publication of the original article [1], we have been notified that Figure 3 was incorrect.

Originally published Figure 3:


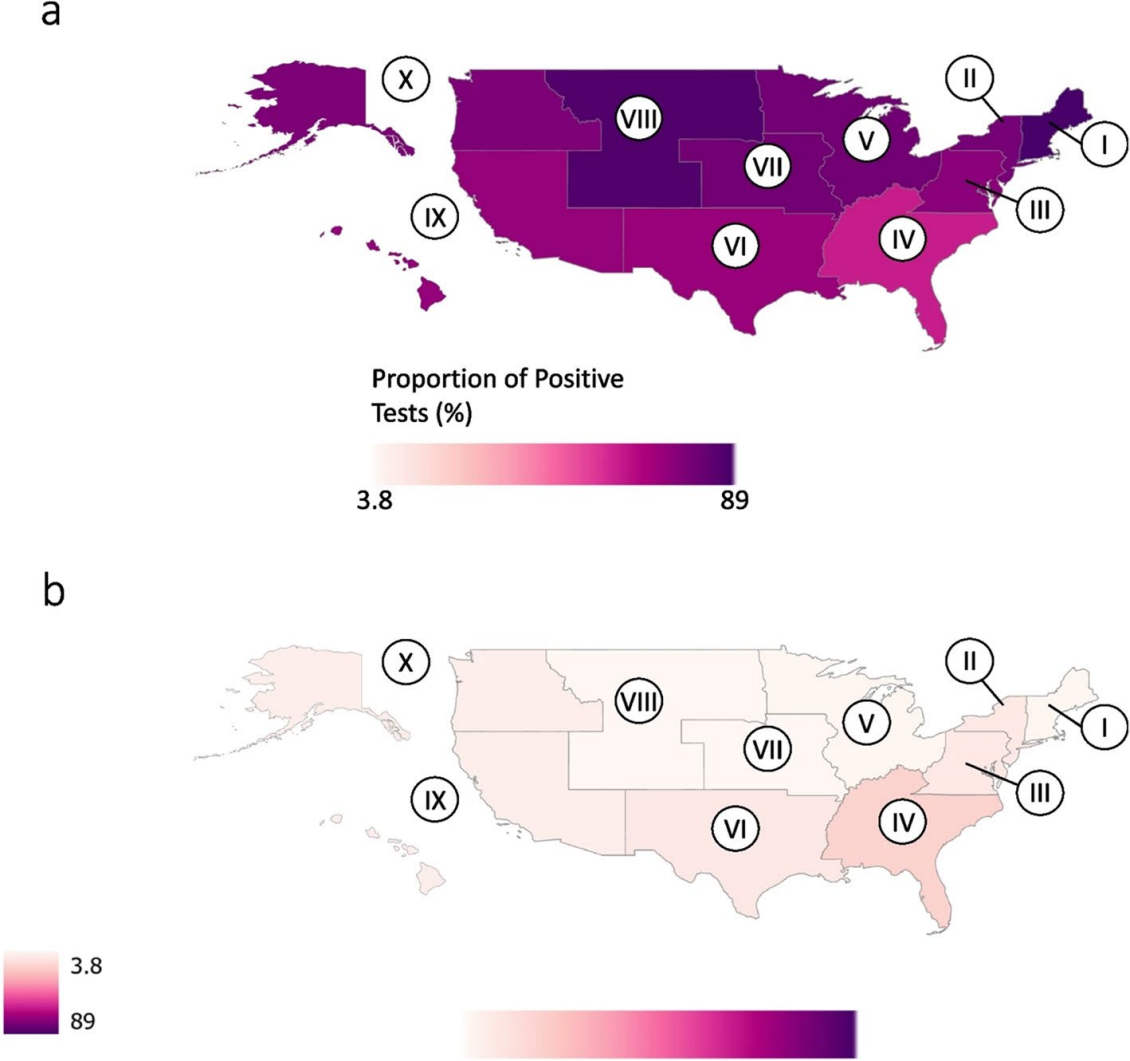


Correct Figure 3:


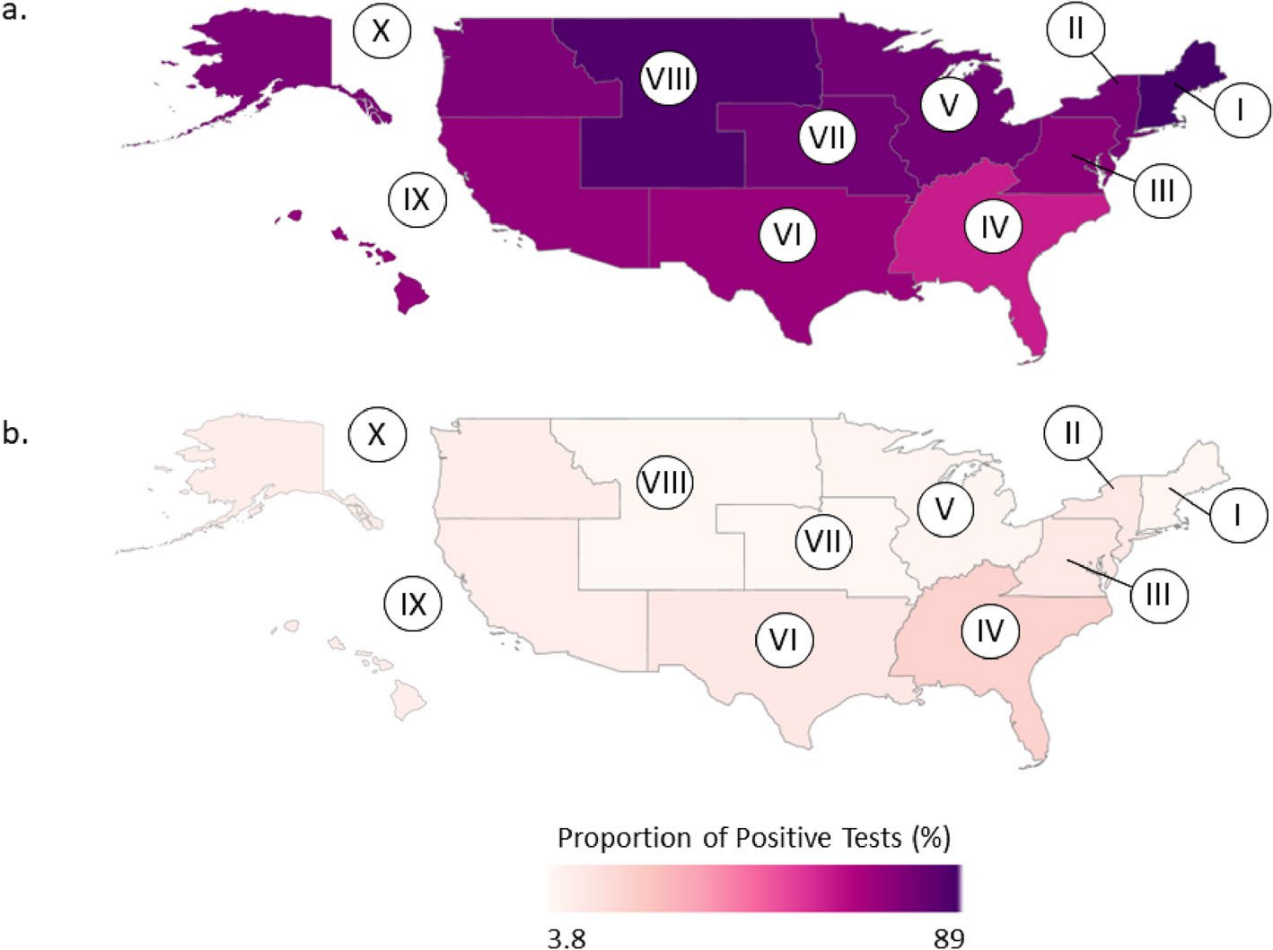


The original article has been corrected.
