# Supplementary material for: Nontuberculous mycobacteria testing and culture positivity in the United States
Source: BMC Infect Dis. 2024 Mar 6;24:288. doi: 10.1186/s12879-024-09059-9 (PMC10916245; doi:10.1186/s12879-024-09059-9)
Supplement: Supplementary file 1 — Additional file 1: Supplemental Figure 1. [file 12879_2024_9059_MOESM1_ESM.docx]

Supplemental Figure 1

| HHS Region | Total Tests (n) | Proportion of Tests (%) |
| --- | --- | --- |
| Region I | 411 | 0.37 |
| Region II | 7703 | 6.8 |
| Region III | 14245 | 12.7 |
| Region IV | 29604 | 26.3 |
| Region V | 5514 | 4.9 |
| Region VI | 15791 | 14 |
| Region VII | 1235 | 1.1 |
| Region VIII | 3895 | 3.5 |
| Region IX | 25932 | 23 |
| Region X | 7992 | 7.1 |
| Other Region | 206 | 0.18 |
| Total | 112528 | 100 |
